# The specificity of action knowledge in sensory and motor systems

**DOI:** 10.3389/fpsyg.2014.00494

**Published:** 2014-05-26

**Authors:** Christine E. Watson, Eileen R. Cardillo, Bianca Bromberger, Anjan Chatterjee

**Affiliations:** ^1^Moss Rehabilitation Research Institute, Einstein Healthcare NetworkElkins Park, PA, USA; ^2^Department of Neurology and Center for Cognitive Neuroscience, University of PennsylvaniaPhiladelphia, PA, USA

**Keywords:** actions, functional magnetic resonance imaging (fMRI), motor system, semantic memory, visual motion

## Abstract

Neuroimaging studies have found that sensorimotor systems are engaged when participants observe actions or comprehend action language. However, most of these studies have asked the binary question of whether action concepts are embodied or not, rather than whether sensory and motor areas of the brain contain graded amounts of information during putative action simulations. To address this question, we used repetition suppression (RS) functional magnetic resonance imaging to determine if functionally-localized motor movement and visual motion regions-of-interest (ROI) and two anatomical ROIs (inferior frontal gyrus, IFG; left posterior middle temporal gyrus, pMTG) were sensitive to changes in the exemplar (e.g., two different people “kicking”) or representational format (e.g., photograph or schematic drawing of someone “kicking”) within pairs of action images. We also investigated whether concrete versus more symbolic depictions of actions (i.e., photographs or schematic drawings) yielded different patterns of activation throughout the brain. We found that during a conceptual task, sensory and motor systems represent actions at different levels of specificity. While the visual motion ROI did not exhibit RS to different exemplars of the same action or to the same action depicted by different formats, the motor movement ROI did. These effects are consistent with “person-specific” action simulations: if the motor system is recruited for action understanding, it does so by activating one's own motor program for an action. We also observed significant repetition enhancement within the IFG ROI to different exemplars or formats of the same action, a result that may indicate additional cognitive processing on these trials. Finally, we found that the recruitment of posterior brain regions by action concepts depends on the format of the input: left lateral occipital cortex and right supramarginal gyrus responded more strongly to symbolic depictions of actions than concrete ones.

## Introduction

A growing body of research suggests that our knowledge about the world is tightly intertwined with the brain's systems for perception and action (Barsalou, 1999; Gallese and Lakoff, 2005; Decety and Grèzes, 2006; see Barsalou, 2008 for a review). On these “embodied” accounts of semantic memory, sensory and motor states from real-world experiences are re-activated, or simulated, when we understand the meaning of words or other symbols (Barsalou, 1999, 2003; Gallese and Lakoff, 2005). In part because of the discovery of neurons in monkeys that fire both during action execution and observation (e.g., Di Pellegrino et al., 1992), researchers have been particularly interested in understanding the way in which the meanings of human actions and events are represented within the semantic system (Pulvermüller, 1999; Vigliocco et al., 2004; Gallese and Lakoff, 2005; Aziz-Zadeh and Damasio, 2008; Gallese and Sinigaglia, 2011). The extant evidence indicates that when we comprehend language referring to actions or think about the actions depicted in photographs or drawings, we engage, at least in part, sensory and motor systems in the brain (e.g., Kable et al., 2002; Hauk et al., 2004; Assmus et al., 2007; Raposo et al., 2009; Saygin et al., 2010). For example, reading words referring to actions performed with different body parts (e.g., “pick,” “lick,” “kick”) activates primary motor and premotor cortex in a somatotopic way (Hauk et al., 2004; see also Boulenger et al., 2009). Similarly, when participants view or make semantic decisions about actions in drawings or photographs (Kable et al., 2002; Assmus et al., 2007), or comprehend sentences describing motion events (Pirog Revill et al., 2008; Saygin et al., 2010 see Gennari, 2012 for a review), activation is observed within area MT+, a part of the visual system specialized for processing motion (Huk et al., 2002). Thus, action concepts may be represented within the same areas of the brain involved in actually executing and perceiving dynamic actions (see Watson et al., 2013 for a meta-analysis of this literature). (Throughout the manuscript, we will use “action concepts” as shorthand for “the semantic representations of actions”.)

However, most studies on the neural basis of action concepts have asked the binary question of whether action concepts are embodied or not, rather than whether action concepts contain graded amounts of sensory and motor information during putative action simulations (see Chatterjee, 2010; Willems and Francken, 2012 for similar critiques). One possible scenario is that action concepts typically evoke the same simulation: different exemplars of an action (e.g., different photographs of someone diving) or different representational formats (e.g., photographs, drawings, or words) produce the same response within sensory and motor systems. Alternatively, neural activity in sensory and motor systems may differ each time an action concept is engaged, preserving details specific to the particular exemplar of an action or format of the input.

In the present study, we addressed this question by examining neural responses to action concepts evoked by different exemplars of actions and by distinct visual formats. First, we used a repetition suppression (RS) paradigm (Grill-Spector and Malach, 2001; Maccotta and Buckner, 2004; Grill-Spector et al., 2006) to determine whether functionally-localized motor movement and visual motion (area MT+) regions-of-interest (ROIs) were sensitive to changes in the exemplar (different people performing the same action) or format (perceptually-rich photographs vs. pared-down, schematic drawings) between pairs of action images. If visual motion or motor areas exhibit decreases in activation (RS) to pairs of images depicting different exemplars of the same action or the same action in different formats, relative to pairs of different action images, it would suggest that an action concept (e.g., running) always evokes the same embodied response. On the other hand, an absence of RS for changes in exemplar or format would be consistent with the hypothesis that sensory and motor simulations preserve instance-specific details about actions.

In addition to these functional ROIs, we also looked for RS within left posterior middle temporal gyrus (pMTG) and bilateral inferior frontal gyri (IFG), two areas of the brain consistently implicated in the representation of semantic knowledge of actions (e.g., Kilner et al., 2009; Kalénine et al., 2010). The proximity of pMTG and IFG to visual motion and motor systems, respectively, enabled us to test the claim that areas of the brain adjacent to modality-specific regions may represent more abstract information derived from those modalities (Plaut, 2002; Thompson-Schill, 2003; Chatterjee, 2008, 2010).

Examining RS within these ROIs allowed us to determine the specificity of action knowledge represented in sensory and motor systems. Additionally, we tested whether photographs of actions and schematic drawings of actions elicited different patterns of activation throughout the brain; we refer to these two types of visual depictions of actions as different “representational formats.” In contrast to perceptually-rich photographs, schematic drawings preserve the fundamental analog structure of the things they represent while eliminating specific perceptual details (Peirce, 1955; Deacon, 1997). As a result, schematic drawings represent meaning more symbolically than photographs, but less symbolically than words. Consequently, schematic drawings may also engage more abstract mental representations than those engaged by concrete percepts, and less abstract representations than those engaged by purely-symbolic language (Chatterjee, 2001). Recent evidence from stroke patients (Amorapanth et al., 2012; Kranjec et al., 2013) implicates the right supramarginal gyrus as harboring such pared-down schematic visual representations.

Additionally, on a graded view of conceptual representation in the brain (Thompson-Schill, 2003; Chatterjee, 2008, 2010), more abstract representations of knowledge are located adjacent to primary sensory and motor cortices. Given that schematic drawings are a more symbolic representational format than photographs, we predict that they will activate brain regions adjacent to those activated by more concrete photographs. Alternatively, areas of the brain involved in representing action concepts may not distinguish between these different representational formats.

## Materials and methods

### Participants

Sixteen participants (7 male; *M*_age_ = 25.3 years, range: 20–34 years) participated in the study. All participants were right-handed, native speakers of English with normal or corrected-to-normal vision and no history of neurologic or psychiatric illness. All participants gave informed consent in accordance with the procedures of the University of Pennsylvania Institutional Review Board and were paid $20/h for their participation. One participant was excluded from the study for having average task accuracy less than 2.5 standard deviations from the group's mean accuracy.

### Stimuli

Stimuli were 30 photographs (hereafter, “pictures”) and 30 schematic drawings (hereafter, “drawings”) of humans performing common transitive or intransitive actions. We created schematic drawings by tracing with a thick red line the configuration of the actor's body in each picture. Drawings of transitive actions contained a simple black shape or line representing the recipient object; drawings of intransitive actions contained a black line representing the ground or other relevant background indicator. To ensure that pictures and drawings were equally recognizable, we collected name agreement measurements from 20 pilot participants. The two image formats did not differ on average name agreement [*M*_pictures_ = 97.9%, *SD*_pictures_ = 2.5; *M*_drawings_ = 97.7%, *SD*_drawings_ = 2.9; *t*_(29)_ = 0.43, *p* > 0.8].

Pictures and drawings depicted six unique actions: three transitive actions (“kick”, “pull”, “push”) and three intransitive actions (“stretch”, “dive”, “walk”). Each action was represented in the stimulus set by five pictures and five corresponding drawings showing different exemplars of the action (e.g., five different people diving).

**Figure 1:**
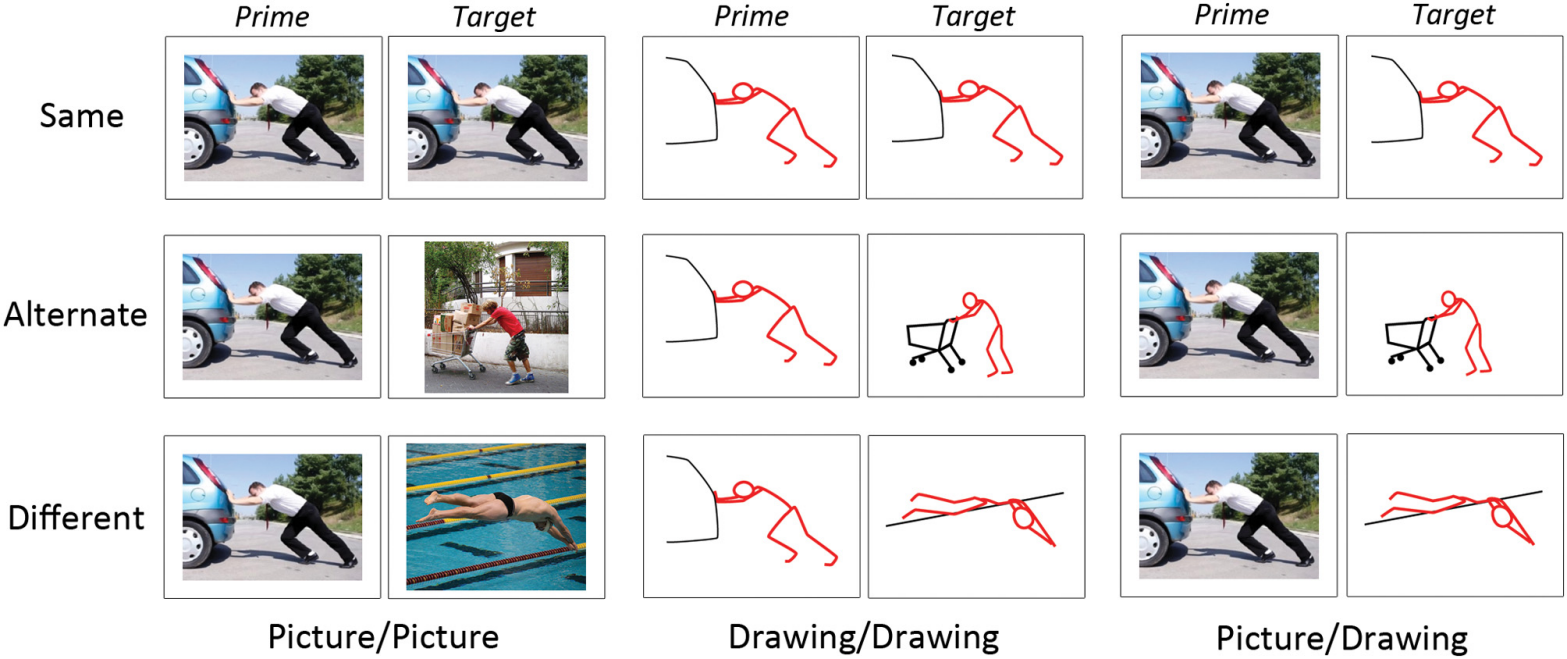
**Examples of experimental stimuli**. Each trial consisted of a prime and target image presented in succession. Images on “Same” trials depicted the same instance of the same action. Images on “Alternate” trials depicted different instances of the same action. Images on “Different” trials depicted different actions. Image pairs were either two photographs (“Picture/Picture”), two schematic drawings (“Drawing/Drawing”), or a photograph followed by a schematic drawing (“Picture/Drawing”).

Each experimental trial contained a prime image and a target image. We paired the 30 pictures and 30 drawings in different ways to form the two conditions of interest (Figure 1). First, we manipulated the representational format of the prime and target (“format type”). The prime and target could both be pictures (Picture/Picture), both drawings (Drawing/Drawing), or the prime could be a picture and the target, a drawing (Picture/Drawing). Critically, we did not examine statistically the fourth combination of format types, Drawing/Picture trials; these trials served as filler trials. We adopted this approach to avoid unnecessarily testing conditions with no unique hypotheses. By examining Picture/Drawing trials, we could assess whether RS occurred between format types. If we used Drawing/Picture trials to address the same question a second time, we would increase the likelihood of a finding a false positive result.

Second, we manipulated the perceptual and/or conceptual similarity between the prime and target (“action similarity”), where “conceptual similarity” refers to the same action (e.g., “kicking”). On “Same” trials, the prime and target depicted the *same exemplar of the same action*; thus, prime and target were similar perceptually and conceptually. On “Alternate” trials, the prime and target depicted *different exemplars of the same action*; thus, the prime and target were similar conceptually but not perceptually. On “Different” trials, the prime and target depicted *different actions* and so were unrelated both perceptually and conceptually. Note that although prime and target were always perceptually similar on Same trials, the degree of this perceptual similarity was greater for Picture/Picture and Drawing/Drawing trials (i.e., the identical picture or drawing as prime and target) relative to Picture/Drawing trials (i.e., the picture and the schematic drawing derived from it as prime and target).

In sum, we manipulated the format type (3) and action similarity (3) of the image pairs. Each cell of our design contained 30 behavioral trials, yielding 270 trials of interest. Given our initial set of 30 pictures and 30 drawings, only 30 prime-target pairings were possible for Same trials of each format type (Picture/Picture, Drawing/Drawing, Picture/Drawing). To create Alternate and Different trials, we selected randomly 30 prime-target pairs from all possible pairings at each level of format type and action similarity. We used these same procedures to select Drawing/Picture filler trials.

### Procedure

During the experiment, participants decided if the prime and target images depicted “the same or different actions” at a conceptual level. The correct response for Same and Alternate trials was “yes” (e.g., prime and target both depict the same exemplar, or different exemplars, of “diving”). The correct response for Different trials was “no” (e.g., prime and target depict “diving” and “kicking”). Prior to entering the scanner, participants completed 5 min of practice trials to ensure that they understood the task. To prevent participants from exploiting low-level visual cues to make their decisions (e.g., correspondences between the image boundaries of prime and target), prime and target images were presented at different random locations on the screen.

On each trial, participants viewed the prime image for 1000 ms, followed by a 250 ms fixation cross. Then, the target image appeared for 1750 ms, during which the participant made his or her response. In total, each trial lasted 3000 ms. On null trials, participants viewed a fixation cross for 3000 ms. Trials were separated by a 500 ms blank screen. The experiment was presented using E-Prime software (Psychology Software Tools, Pittsburgh, PA) on a computer connected to a projector. Manual responses and reaction times (RTs) were recorded with a button box held by participants with both hands. “Yes” or “no” responses were made by pressing a button with the left or right thumb. Half of the participants indicated “yes” responses with a right button press and “no” responses with a left button press; the other half of participants were assigned the reverse pattern. While in the scanner, participants completed 270 trials of interest, 90 filler trials, and 90 null trials. Trials were presented in five scanning runs of 5.4 min each. Each run began with 9 s of introductory screens. Following these “ready screens,” experimental, filler, and null trials occurred randomly within and across runs for each participant.

After the experimental trials, participants completed two functional localizer scans. During the visual motion (area MT+) localizer, participants passively viewed four 32.5-s blocks each of moving (flow fields) or stationary white dots on a black background (Bavelier et al., 2001; Saygin et al., 2010). During the motor movement localizer, participants were instructed via computer screen to move the right hand, left hand, right foot, and left foot continuously for 20 s, or to rest for 20 s (Hauk et al., 2004; Boulenger et al., 2009; Raposo et al., 2009). Each type of block was presented 4 times.

### Data acquisition

We collected structural and functional data on a 3.0 Tesla Siemens Trio scanner using an eight-channel head coil. We acquired high-resolution T1-weighted structural images using a MP-RAGE pulse sequence and near-isotropic voxels (0.98 × 0.98 × 1 mm). T2^*^-weighted echo-planar images were collected during the five experimental scanning runs (104 volumes each), the MT+ localizer (91 volumes), and the motor localizer (102 volumes) (repetition time = 3 s; echo time = 30 ms; flip angle = 90°; field of view = 220 mm; slice thickness = 3 mm; matrix size = 64 × 64; voxel size = 3.4 × 3.4 × 3 mm). Each functional volume consisted of 50 axial slices that covered the whole cerebral cortex.

### fMRI data preprocessing

Imaging data was preprocessed and analyzed using the FMRIB Software Library (FSL version 4.1; http://www.fmrib.ox.ac.uk/fsl). The first three volumes of each functional run were discarded to allow for steady state magnetization. Functional data were slice timing corrected using sinc interpolation, motion corrected, and high-pass filtered (0.01 Hz). For each participant, functional data from each run were registered to a participant's high-resolution structural image using FMRIB's Linear Registration Tool with 7° of freedom. One set of functional data for use in region-of-interest analyses was kept in each participant's native space and smoothed with a Gaussian kernel of 4 mm (full-width at half-maximum). A second copy of functional data for use in group-level analyses was registered to Montreal Neurological Institute standard space (MNI-152) using linear registration with 12° of freedom and smoothed with a Gaussian kernel of 8 mm.

### First-level analyses

We first modeled each functional scanning run separately for each participant with FMRIB's FEAT (fMRI Expert Analysis Tool). We used an event-related model in which the events of interest began with the onset of the prime image and ended with the offset of the target image. Events were modeled as single impulses convolved with FSL's double-gamma hemodynamic response function (HRF), along with the event's temporal derivative. Regressors were created for each format type/action similarity combination [e.g., Picture/Picture(Same), Picture/Picture(Alternate), etc.], and for filler trials and null trials. Contrasts of interest were computed at the first level using linear combinations of these regressors.

### Higher-level analyses

For each participant, contrasts between conditions modeled within a run were combined at the second-level using a fixed effects model within FMRIB's Local Analysis of Mixed Effects (FLAME). Finally, contrasts intended for third-level, group analyses were combined across participants using a mixed effects model (FLAME1+2). Resulting group-level maps of *z*-statistics were thresholded at *z* > 2.3 with a corrected cluster significance threshold of *p* < 0.05 (Worsley et al., 1992). In order to compare the location of the visual motion ROI with our group-level results, we also computed the location of the visual motion ROI at the group level. To more precisely determine the anatomical location of this region, we thresholded this analysis using voxel-based, rather than cluster-based, thresholding (GRF-theory-based maximum height thresholding with *p* < 0.05, corrected) (Worsley et al., 1992).

### Region-of-interest analyses

For region-of-interest (ROI) analyses, we used FMRIB's Featquery tool to compute, for each participant, the mean contrast of parameter estimates in each ROI for each condition [i.e., Picture/Picture (Same), Picture/Picture (Alternate), etc.] minus null (fixation) trials. With this data, within-subject RS effects were evaluated using SPSS software. We looked for RS within each ROI by looking for effects of action similarity (Same, Alternate, Different) and format type (Picture/Picture, Drawing/Drawing, Picture/Drawing) using a two-way repeated measures ANOVA. When we observed an interaction between action similarity and format type, *p*-values from tests of simple effects were corrected for multiple comparisons using the Holm-Sidak method.

Our two ROIs of primary interest were defined functionally for each participant. Visual motion ROIs were defined by contrasting blocks in which participants perceived moving vs. stationary dots (see above). The resulting map of *z*-values for this contrast was thresholded first at a False Discovery Rate (FDR) (Nichols and Holmes, 2002) of *q* = 0.000001. (Here, we used the FDR method given that it controls the family-wise error rate without being overly conservative for low smoothness data with few degrees of freedom, Nichols and Hayasaka, 2003.) We then selected the largest cluster in each hemisphere that survived this threshold and fell within lateral occipital cortex. This anatomical constraint was applied rarely and excluded clusters that emerged in the occipital poles. Using this procedure, visual motion ROIs were localized for 10 participants. For 2 participants, no voxels survived at this threshold, so we used a more lenient threshold of *q* = 0.05. We note that using a more lenient threshold to identify ROIs in some participants does not bias us to find differences between the experimental conditions. On the contrary, by using voxels that respond less strongly to visual motion, we may have increased noise in our analyses, making it more difficult to detect effects. For 3 participants, no visual-motion-preferring voxels were detected even at a relaxed threshold. The average visual motion ROI had a volume of 7995 mm^3^ (*SD* = 5420).

Motor movement ROIs were defined in each participant by contrasting the movement of each effector (left hand, right hand, left foot, right foot) with rest (see above). Resulting *z*-maps for each of these contrasts were thresholded with the same general procedure described for the visual motion ROI. For each effector, we selected the largest cluster that survived the threshold. Clusters for each of the four effectors were then combined to form a participant's entire motor movement ROI. In 10 participants, a motor ROI was identified at *q* = 0.0000001; for 2 other participants, the threshold was relaxed to *q* = 0.05. We were unable to identify a motor movement ROI in 3 participants. The average motor movement ROI had a volume of 22813 mm^3^ (*SD* = 11111).

**Figure 2:**
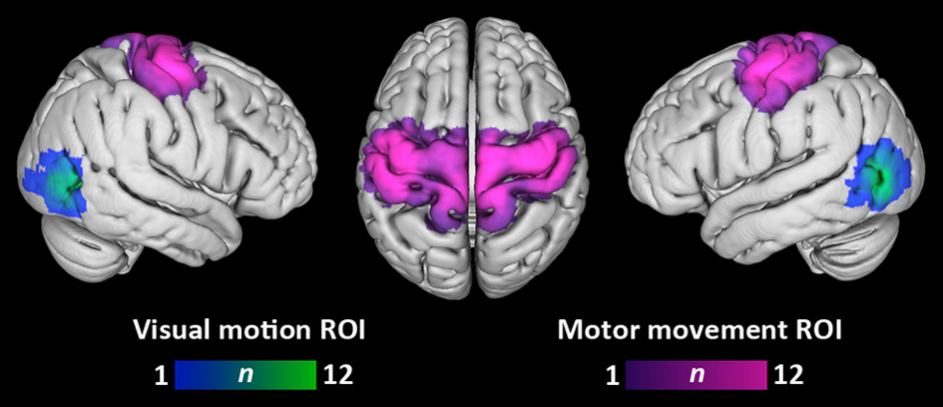
**Overlap of visual motion and motor movement regions-of-interest across participants**. Each participant's ROIs have been transformed into standard MNI space. Color bars denote the number of participants having a given ROI at each voxel. Overlap is displayed at a search depth of 3 mm.

Figure 2 depicts the overlap of participants' visual motion and motor movement ROIs transformed into MNI-152 standard space. The location of visual motion ROIs within lateral temporo-occipital cortex agrees with previous localizations of area MT+ (e.g., Dumoulin et al., 2000). Motor movement ROIs primarily covered lateral and medial pre- and post-central gyri.

To ensure that RS within the motor movement ROI could not be attributed to lower-level processes, we made a further adjustment to analyses performed within each participant's motor movement ROI. In the experimental task, trials on which a participant responds “yes” (i.e., Same and Alternate trials) occurred more frequently than “no” trials (i.e., Different trials). Since participants used one hand more often throughout the experiment, it is possible that we could observe a decrease in neural activity for Same/Alternate trials relative to Different trials within motor regions due to manual response priming (i.e., repeated use of one hand for responding). Therefore, we calculated the effects of Same/Alternate trials (relative to null trials) and Different trials (relative to null trials) *only* within the hemisphere ipsilateral to the manual response for each condition. In other words, for analyses within the motor movement ROI, we only considered activation within the hemisphere not responsible for a participant's button press. For participants who responded “yes” with the right hand (to Same/Alternate trials), mean contrast of parameter estimates for Same and Alternate trials relative to null were computed only within the right hemisphere motor movement ROI; mean contrast of parameter estimates for Different trials (“no” responses made with the left hand) were computed only within the left hemisphere motor movement ROI. In using this procedure, we ensured that RS effects observed within motor regions could be attributable only to the experimental manipulations rather than priming of manual responses.

In addition to these two functionally-defined ROIs, we created two anatomical ROIs: bilateral IFG and left pMTG. Each area was taken from the Harvard-Oxford Cortical Atlas that is registered to MNI-152 standard space and included in the FSL distribution. ROIs in standard space were transformed into each participant's native space using linear registration (FLIRT). For each ROI, we excluded any voxels that were also included in a participant's functionally-defined visual motion and motor movement ROIs to ensure that observations within the ROIs were independent of each other. Similarly, participants for whom visual motion (*n* = 3) and motor movement (*n* = 3) ROIs could not be located were excluded from IFG and pMTG ROI analyses given that we could not rule out overlap between functionally-responsive and anatomically-localized areas in these participants. Finally, given the contribution of IFG to action execution (e.g., Caspers et al., 2010; Press et al., 2012), we analyzed activation with the IFG ROI in the same manner as the motor movement ROI (see above).

## Results

### Behavioral analyses

**Figure 3:**
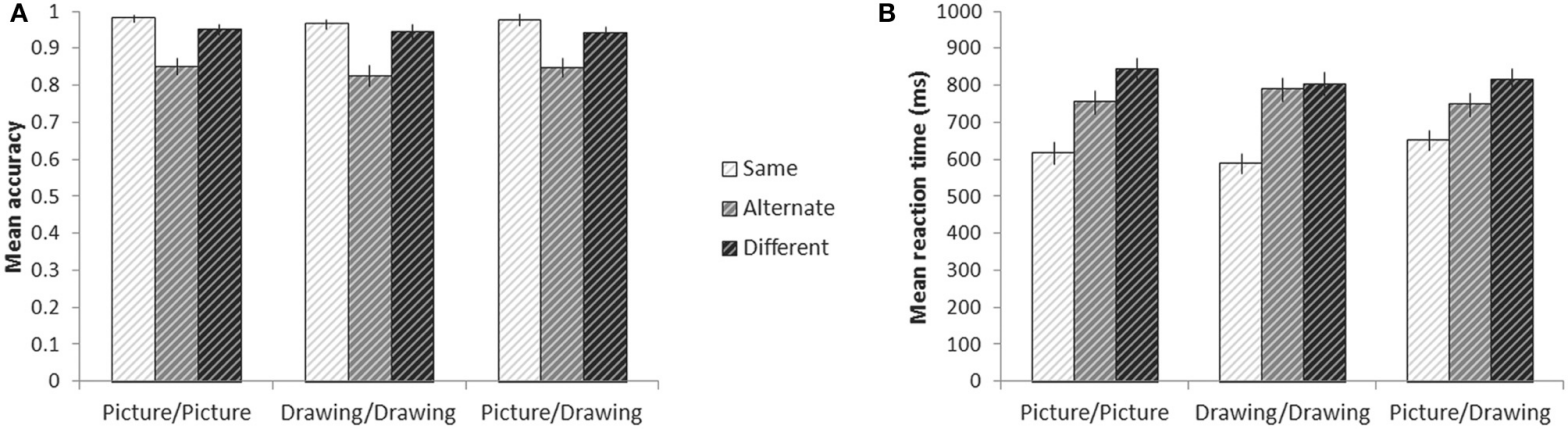
**Behavior on the experimental tasks while in the scanner**. Mean accuracy **(A)** and reaction time **(B)** for each condition. Error bars denote plus or minus one standard error of the mean.

We used a two-way repeated measures ANOVA to look for effects of action similarity (Same, Alternate, and Different) and format type (Picture/Picture, Drawing/Drawing, and Picture/Drawing) on accuracy. We found a significant effect of action similarity [*F*_(2, 28)_ = 28.7, *p* < 0.001] and a marginal effect of format type [*F*_(2, 28)_ = 2.7, *p* = 0.08] (Figure 3A). The interaction between action similarity and format type was not significant. Pairwise comparisons revealed that participants were significantly less accurate on Alternate trials relative to Different (*p* = 0.02) and Same (*p* = 0.02) trials, and significantly less accurate on Different trials than Same trials (*p* = 0.01). Pairwise comparisons between format types showed that participants were significantly less accurate on Drawing/Drawing trials than Picture/Picture trials (*p* = 0.03); however, the mean difference in accuracy between these conditions was very small (1.6%). No other pairwise differences between format types reached significant.

Reaction time analyses were conducted only for correct trials. There was a significant effect of action similarity on participants' RTs [*F*_(2, 28)_ = 67.2, *p* < 0.001] and a significant interaction between action similarity and format type [*F*_(4, 56)_ = 30.0, *p* < 0.001] (Figure 3B). The effect of format type was not significant. To explore the interaction, we calculated simple effects between levels of action similarity for each format type. For every format type, participants responded to Same trials significantly faster than either Alternate trials (all *p* < 0.001) or Different trials (all *p* < 0.001). For Picture/Picture trials, participants also responded more quickly to Alternate trials than Different trials (*p* = 0.005). For Drawing/Drawing and Picture/Drawing trials, however, there was no significant difference between RTs to Alternate and Different trials. When jointly considering participants' RTs and accuracy, we note that participants' lower accuracy on Alternate trials may not reflect errors, *per se*, but individual differences in whether a participant believed the two images indeed depicted the same action. On the other hand, reaction time analyses were only carried out on trials in which participants accepted identical and alternate exemplars and rejected images of different actions as depicting the same action; RTs thus reflect the time to accumulate sufficient information to make each type of decision (e.g., Ratcliff, 1978).

### ROI analyses

**Figure 4:**
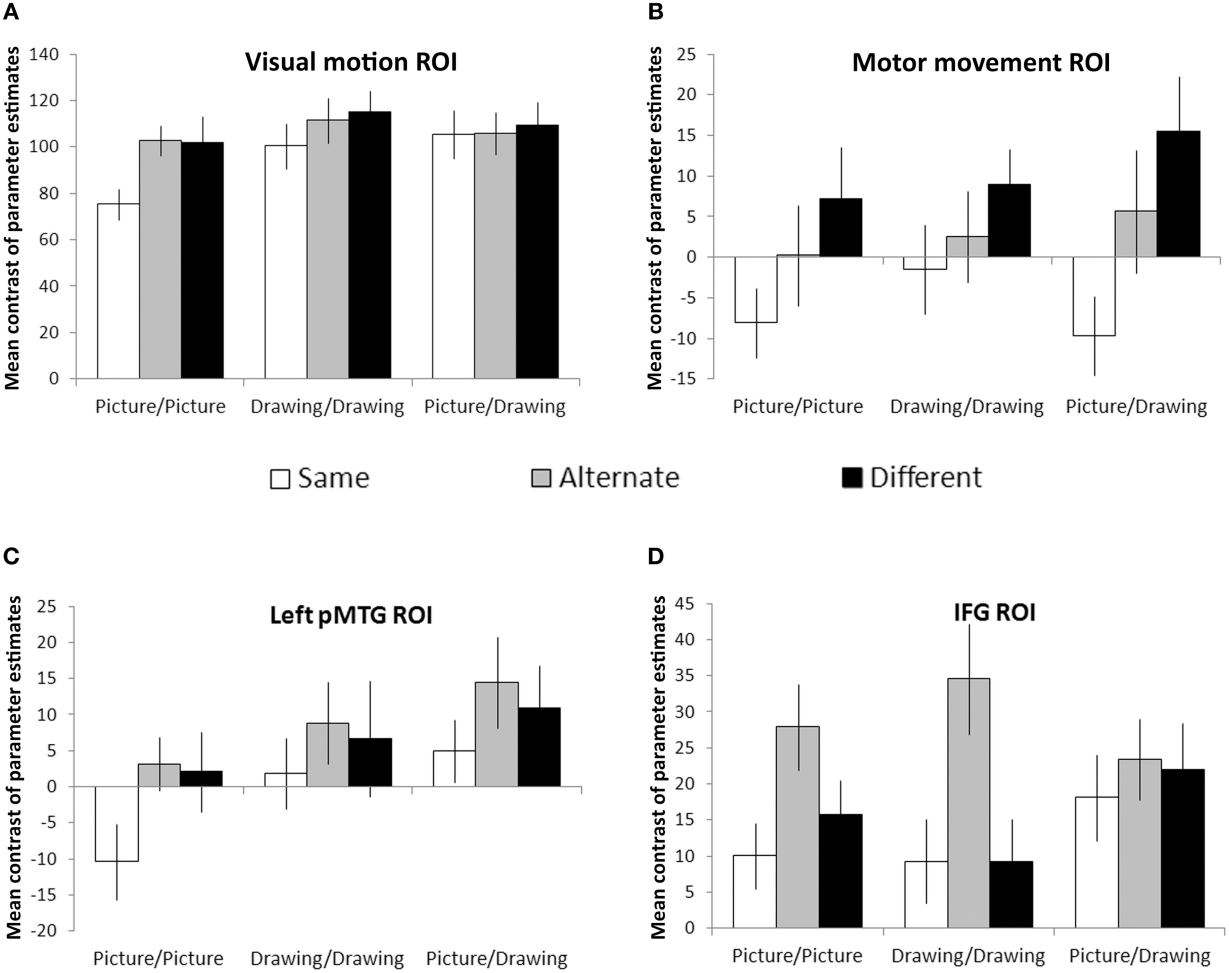
**Region-of-interest analyses**. Visual motion **(A)** and motor movement **(B)** areas were functionally-localized in each participant. Left pMTG **(C)** and bilateral IFG **(D)** were defined anatomically using the Harvard-Oxford cortical atlas. Bars reflect the mean contrast of parameter estimates between each condition and null (fixation) trials. Error bars denote plus or minus one standard error of the mean.

Visual motion and motor movement ROIs were functionally-localized for each participant. For each participant, we calculated the mean contrast of parameter estimates between each condition and null (fixation) trials within these regions. Then, we looked for effects of the action similarity (Same, Alternate, Different) and format type (Picture/Picture, Drawing/Drawing, and Picture/Drawing) of the prime and target images using a two-way repeated measures ANOVA. Within the visual motion ROI, there were significant effects of action similarity [*F*_(2, 22)_ = 8.3, *p* = 0.002] and format type [*F*_(2, 22)_ = 7.0, *p* = 0.005], and a marginally significant interaction between the two [*F*_(4, 44)_ = 2.2, *p* = 0.08] (Figure 4A). Simple effects between levels of action similarity for each format type showed significant suppression for Same trials relative to Different (*p* = 0.03) and relative to Alternate (*p* = 0.003) trials only for the Picture/Picture condition. No other pairwise comparisons were significant. Thus, the visual motion ROI exhibited RS only when the prime and target images were identical, perceptually-rich photographs of actions.

We evaluated RS effects within the motor movement ROI only within the hemisphere ipsilateral to each condition's expected manual response (see Materials and Methods). We observed a significant effect of action similarity [*F*_(2, 22)_ = 8.4, *p* = 0.002] but no effect of format type or interaction between the two (Figure 4B). Planned comparisons between each level of action similarity showed significant suppression for Same trials relative to Different (*p* = 0.006) and Alternate trials (*p* = 0.01). Suppression for Alternate trials relative to Different trials was not significant but showed a trend in that direction (*p* = 0.09). However, the main effect of action similarity was significantly fit by a linear contrast between Same, Alternate, and Different levels [*F*_(1, 22)_ = 11.5, *p* = 0.006], suggesting that RS occurred in the motor movement ROI when the prime and target images referred to the same basic action, even if different exemplars or representational formats.

Next, we looked for effects of action similarity and format type within areas of the brain near to functionally-localized visual motion and motor movement ROIs. Within left pMTG, we observed significant effects of format type [*F*_(2, 22)_ = 9.5, *p* = 0.001] and action similarity [*F*_(2, 22)_ = 3.8, *p* = 0.04], but no significant interaction between the two (Figure 4C). Planned comparisons between each level of action similarity revealed significant suppression for Same trials relative to Alternate trials (*p* = 0.03) and marginally significant suppression for Same trials relative to Different trials (*p* = 0.08). There was no difference between Alternate and Different trials. Planned comparisons between each format type indicated significantly less activation within left pMTG for Picture/Picture trials relative to Drawing/Drawing (*p* = 0.01) or Picture/Drawing trials (*p* = 0.001), and Drawing/Drawing and Picture/Drawing trials were not significantly different from one another. Thus, left pMTG exhibited suppression when the prime and target were identical but not when they were merely different exemplars of the same action. And, this area of the brain was more strongly activated overall when the prime or target image was a schematic drawing of an action.

Finally, we examined RS effects within the IFG. As with the motor movement ROI, we analyzed activation within the hemisphere ipsilateral to each condition's expected manual response (see Materials and Methods). Within IFG, we found a significant effect of action similarity [*F*_(2, 22)_ = 8.1, *p* = 0.002]. There was no effect of format type or interaction (Figure 4D). Planned comparisons between levels of action similarity revealed no difference between activation on Same and Different trials (*p* = 0.53). Surprisingly, we also observed significant enhancement (i.e., an increase) for Alternate trials relative to both Different (*p* = 0.02) and Same (*p* < 0.001) trials. This result indicates that IFG exhibited not suppression, but *increased* activity when the images depicted different exemplars of the same action.

Although these analyses examined the *patterns* of RS effects between conditions, we note that the overall magnitude of values within each ROI reflects the degree to which an ROI was more active during the task than fixation. For example, large mean contrasts of parameter estimates within the visual motion ROI likely reflect the richer visual input present on experimental trials relative to fixation crosses.

### Whole-brain analyses

**Figure 5:**
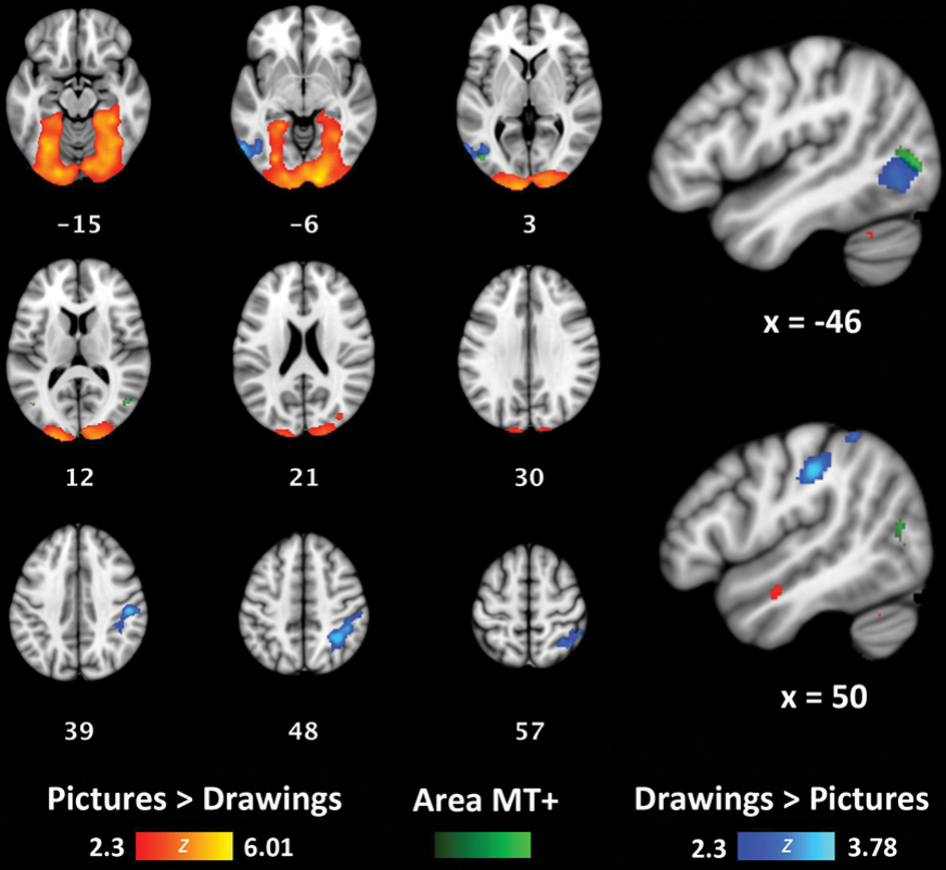
**Whole-brain analyses contrasting Picture/Picture(Different) (red/yellow) and Drawing/Drawing(Different) (blue/light blue) trials**. The group-level location of visual-motion-preferring voxels is shown in green. Coordinates reported in MNI standardized space.

To determine if concrete and more symbolic representations of actions activate distinct areas throughout the brain, we also used a whole-brain, group-level analysis to compare activation for perceptually-rich photographs of actions (Picture/Picture trials) with activation for schematic drawings of actions (Drawing/Drawing trials). Because Same and Alternate trials were hypothesized to exhibit RS effects, we only compared Different trials for each of these two formats. Relative to Drawings, Pictures activated a large, bilateral cluster that began in the occipital poles and extended into the fusiform gyri in both hemispheres (volume = 32710 mm^3^; maximum *z*-value = 6.01; MNI coordinates of maximum: *x* = 16, *y* = −96, *z* = −8) (Figure 5, red/yellow). Relative to Pictures, Drawings activated a cluster in the right supramarginal gyrus and superior parietal lobule (volume = 3096 mm^3^; maximum *z*-value = 3.78; MNI coordinates of maximum: *x* = 32, *y* = −52, *z* = 52) (Figure 5, light blue/dark blue). Drawings also activated a smaller cluster within left lateral occipital cortex (volume = 1782 mm^3^; maximum *z*-value = 3.51; coordinates of maximum: *x* = −58, *y* = −66; *z* = −6). The majority of voxels in this cluster were located anterior to the typical location of area MT+, as reported in other studies (Dumoulin et al., 2000) and within our own participant group (Figure 5, group-level visual-motion-preferring voxels shown in light green/dark green).

## Discussion

In the present study, we used RS fMRI to determine the specificity of information carried by sensory and motor systems during conceptual processing of actions. Of primary interest was whether brain regions involved in performing movements and perceiving visual motion, two areas of the brain often engaged by action concepts (Hauk et al., 2004; e.g., Kable et al., 2002), were sensitive to changes in the exemplar or representational format of pairs of action images.

Our results reveal strikingly different response patterns between these two brain areas: while the visual motion ROI exhibited RS only for identical photographs of actions, suppression occurred in the motor movement ROI for repetitions of the same *and* alternate exemplars of an action, irrespective of the format. This result suggests that neural activity within these sensorimotor regions during semantic tasks represents information about actions at different levels of specificity. On the one hand, during comprehension of static depictions of actions, voxels that respond strongly to visual motion appear to encode information highly specific to a particular exemplar of an action or particular representational format: only when the prime and target images were identical *and* conveyed many perceptual details about the actor or action context did we observe RS within the visual motion ROI. Because this region was strongly active for all conditions, it cannot be the case that some conditions merely failed to activate visual motion areas at all. Instead, neural responses to action concepts within this area preserve detailed information about the specific instance of an action; different actors and/or representational formats activate different neural representations. Furthermore, we did not observe RS when prime and target images were identical schematic drawings. Thus, the absence of perceptually-rich details in schematic drawings may result in a more variable response within areas specialized for visual motion, even across repeated instances of the same schematic drawing.

Although we focused on the activation of visual motion areas by conceptual processing of *static* action images, our results accord with other studies on the response of area MT+ to different types of visual motion. In particular, this area is sensitive to changes in the speed, direction, and velocity of low-level visual motion (Wall et al., 2008; Lingnau et al., 2009; Cardin et al., 2012; Weigelt et al., 2012). Thus, to the extent that different exemplars of an action or different representational formats convey actions performed at different speeds, in different directions, etc., the response within visual motion regions may differ.

Yet, our results are at odds with two prior studies investigating RS between pairs of *dynamic* action stimuli (i.e., videos) using a semantic task (Kable and Chatterjee, 2006; Wiggett and Downing, 2010; but see Grossman et al., 2010). In both of these studies, area MT+ was insensitive to changes in the actor and thus responded similarly as long as the same action was repeated (e.g., “kicking”). Given that both of these studies used stimuli that contained actual visual motion, an alternative explanation of the present results is that area MT+ exhibits a narrower range of responses to static images than dynamic action stimuli. Although static images engage this area, they may do less strongly and with less variability than dynamic depictions of actions. If so, then the absence of RS to alternate exemplars within the visual motion ROI in the current study may reflect insufficient physiological power to detect differences between all conditions in this area.

In contrast to the highly-specific effects we observed within the visual motion ROI, the motor movement ROI exhibited RS between pairs of images that depicted identical actions *and* pairs that depicted alternate exemplars of the same action. This response occurred both when the prime and target were the same format (Picture/Picture, Drawing/Drawing) or different formats (Picture/Drawing). This result suggests that a similar representation is evoked within the motor system irrespective of the way in which an action concept is accessed; the same motor simulation is produced in response to different exemplars of the same action or to actions presented in different formats.

One way in which this result could arise is if motor simulations are grounded in person-specific motor programs for actions. In other words, no matter who I perceive doing an action (e.g., Jack kicking, Jane kicking) or the format of the input (e.g., a photograph or schematic drawing of “kicking”), my motor simulation will reflect the way in which *I* am inclined to kick. Indeed, there is prior evidence that the involvement of motor regions in representing action concepts depends on an individual's particular physical experiences (Calvo-Merino et al., 2005, 2006; Beilock et al., 2008). For example, Calvo-Merino et al. (2005) found that the degree to which expert ballet and capoeira dancers recruited motor regions during action observation differed when watching their own style of dance versus the other; the authors conclude that “… action observation evokes individual, acquired motor representations… ” (p. 1247). Similarly, participants' ability to recall actions depends on their motor expertise with those actions (Pezzulo et al., 2010). The present results extend these findings by suggesting that an action evokes the same person-specific motor simulation irrespective of the way in which an action concept is accessed.

However, we note that the degree to which the motor system participates in representing action concepts *at all* is also modulated by physical experience (described above) and task demands (Van Dam et al., 2012). Our recent meta-analysis of neuroimaging studies using action words and action images did not find consistent involvement of premotor or primary motor cortex in conceptual processing of these stimuli (Watson et al., 2013). In the current study, we used a small set of very familiar actions, and we functionally-localized areas involved in performing movements within each participant. Therefore, we may have been more likely than other studies to generate and detect effects within the motor system during conceptual processing of actions.

Even though participants made manual responses on each trial, our study design makes it unlikely that the RS we observed within the motor movement ROI reflects manual response priming. First, for each participant, we only analyzed activation within the hemisphere that was ipsilateral to each condition's expected response. Thus, results from the motor movement ROI reflect activation within the hemisphere not responsible for the button press. Second, the RS effects were not entirely determined by activation within hand-preferring parts of the motor system: we also functionally-localized areas active when performing foot movements. Finally, we observed significantly different levels of activation within the motor movement ROI for Same and Alternate trials. If manual response priming was driving suppression effects, then we would expect *no* difference between conditions responded to with the same hand.

We used functionally-defined visual motion and motor movement ROIs rather than ROIs defined anatomically or from group-level results. However, since the tasks used to define these ROIs did not require measurable behavioral responses, we cannot be certain that a given participant was paying attention or performing the localizer task; indeed, differences in task engagement may explain why visual motion and motor movement ROIs could not be identified, or required a more lenient threshold to be identified, in some participants. Yet, given the potentially variable functional brain organization of each participant, using ROIs defined in this way allowed us to more precisely test functionally-motivated hypotheses (see Saxe et al., 2006 for a similar argument), i.e., that voxels that participate in more basic cognitive tasks (processing visual motion, executing body movements) would encode information at different levels of specificity during a conceptual task.

We also examined RS effects in anatomically-defined ROIs. Within two brain areas neighboring visual motion and motor movement ROIs, we observed RS when the prime and target image depicted the same instance of the same action, but not different instances of the same action. Instead, within left pMTG, we observed no differentiation between Alternate and Different trials, and within IFG, we observed *enhancement* for Alternate relative to Different and Same trials. In some respects, these results are surprising: some researchers have suggested a “graded” view of embodiment in which more abstract representations of action meaning are represented in brain areas adjacent to modality-specific cortices (Thompson-Schill, 2003; Kable et al., 2005; Chatterjee, 2008, 2010). Therefore, we expected to observe RS for different exemplars of the same action within left pMTG and IFG. However, our pattern of results may be consistent with findings of “repetition enhancement” rather than “repetition suppression” (Raposo et al., 2006; Kuperberg et al., 2008; see Segaert et al., 2013 for a review). One hypothesis is that while suppression occurs when the same cognitive process is performed on a prime and target, enhancement occurs when the target requires additional processes, like explicit memory retrieval (Henson, 2003).

In the current study, we found significant enhancement for Alternate trials within IFG and non-significant but numerically higher activation for Alternate trials relative to Different trials for each format type within left pMTG. Alternate trials were also the most difficult for participants. Therefore, it is possible that verifying alternate exemplars of the same action (vs. the easier tasks of verifying an identical match or a complete mismatch) required additional cognitive processing—and neural activity—within IFG and left pMTG. IFG, in particular, has been shown to play a role in selecting among competing representations in memory (Thompson-Schill et al., 1997; Moss et al., 2005). When determining whether two images were different exemplars of the same action, participants may have had to exert more cognitive effort to find the link between two conceptually similar, but perceptually dissimilar, instances of an action. Lack of RS and numerical enhancement within pMTG may similarly reflect participants' greater need to retrieve explicit information about actions in the Alternate condition.

Finally, we investigated at the whole-brain level the degree to which the brain distinguishes between perceptually-rich photographs of actions and more symbolic schematic drawings of actions. Given that they contain more visual details than drawings, pictures unsurprisingly yielded greater activation throughout early visual cortex. The reverse comparison, however, yielded greater activation for schematic drawings in two areas of the brain. First, drawings more strongly engaged the right supramarginal gyrus and parts of the superior parietal lobe, a result in agreement with a recent voxel-based lesion-symptom mapping (VLSM) study from our lab. In this study, stroke patients with damage to the left or right hemisphere matched categorical spatial relations among objects (e.g., “above,” “below”) across different representational formats (i.e., pictures, schematic drawings, and words) (Amorapanth et al., 2012). Patients with damage to right supramarginal gyrus were particularly impaired matching spatial relation words to their corresponding schematic drawings relative to their corresponding pictures. A recent case study also supports the view that schematic drawings are processed differently than perceptually-rich photographs: a patient with simultagnosia, a condition in which patients are characteristically unable to perceive more than a single object at a time (Luria, 1959), was better able to comprehend spatial relations between objects (e.g., “above,” “below”) when they were depicted as schematic drawings rather than as photographs (Kranjec et al., 2013). Given the present results as well as neuroimaging evidence for the activation of right supramarginal gyrus during the naming of spatial relations between objects (e.g., Damasio et al., 2001), this part of the brain may be responsible for recognizing the schematic structure of these pared-down percepts.

We also found greater activation for schematic drawings of actions relative to photographs in left lateral occipital cortex; most voxels in this cluster were located anterior to visual motion-preferring areas, in lateral occipital cortex and the most posterior aspect of pMTG. This result is consistent with a graded view of conceptual representation (Chatterjee, 2008, 2010; Watson and Chatterjee, 2011). Action knowledge derived from visual motion area MT+ is represented along a temporal posterior-to-anterior axis in which increasingly abstract information is represented more anteriorly. Accordingly, a brain area anterior to area MT+ responded more strongly to pared-down, more symbolic schematic drawings than to perceptually-rich photographs of actions. We also observed greater overall activation of the left pMTG ROI for trials that included a schematic drawing (Picture/Drawing or Drawing/Drawing trials). Together, these results suggest that more abstract or symbolic depictions of actions recruit areas adjacent to modality-specific cortices. Consistent with this claim, we found using a meta-analysis approach that words referring to actions consistently activated an area within left middle temporal gyrus anterior to the area associated with visual depictions of actions (Watson et al., 2013). The implication of these findings for embodied accounts of semantic knowledge is that the recruitment of modality-specific—or other—regions depends on whether concepts are accessed by more or less symbolic means. More symbolic depictions may additionally, or instead, recruit information that is abstracted from direct experience and represented adjacent to modality-specific areas.

Finally, we acknowledge that participants' did not *need* to access conceptual knowledge of actions on all trials. When the prime and target images were identical (Same trials), participants' decisions could be based solely on visual similarity. We note that the RS effects seen in the visual motion ROI suggest that some inference about the images is being made even when they are perceptually identical insofar as neural activity in an area sensitive to visual motion is influenced by static images. A visual similarity strategy would not work on the Alternate and Different trials: though prime and target stimuli were visually dissimilar for both, these trial types required different behavioral responses. Therefore, participants' needed to access the *meaning* of the actions depicted in these images in order to make a response. Furthermore, the pattern of results suggests that participants drew upon action concepts even on Same trials: it is not obvious why the repetition of visually similar images should yield decreased activation in the motor movement ROI. Instead, we suggest that the conceptual similarity of these images—and images in the Alternate condition—produces RS within the motor movement ROI.

Understanding the specificity of brain regions to different exemplars of actions and representational formats makes embodied accounts of the semantic system more precise. Here, we found that sensory and motor systems carried different amounts of information during conceptual processing of actions: while visual motion areas preserved exemplar- and format-specific details, regions involved in performing movements responded similarly as long as images referred to the same basic action (e.g., “kicking”). Thus, when the motor system participates in understanding an action, it may do so by activating one's own motor program for that particular action. Additionally, two brain regions (left lateral occipital cortex and right supramarginal gyrus) responded more strongly to more symbolic representations of actions (i.e., schematic drawings) than to concrete ones (i.e., photographs). For embodied accounts, these data indicate that even outside of area MT+, the recruitment of posterior brain regions by action concepts depends on the format of the input. Within lateral occipitotemporal cortex, in particular, more abstract representations of actions may be represented adjacent to modality-specific cortical areas.

### Conflict of interest statement

The authors declare that the research was conducted in the absence of any commercial or financial relationships that could be construed as a potential conflict of interest.
